# Supplementary figures and images for: Seeing what you hear: Compression of rat visual perceptual space by task-irrelevant sounds
Source: PLoS Comput Biol. 2025 Oct 29;21(10):e1013608. doi: 10.1371/journal.pcbi.1013608 (PMC12571299; doi:10.1371/journal.pcbi.1013608)

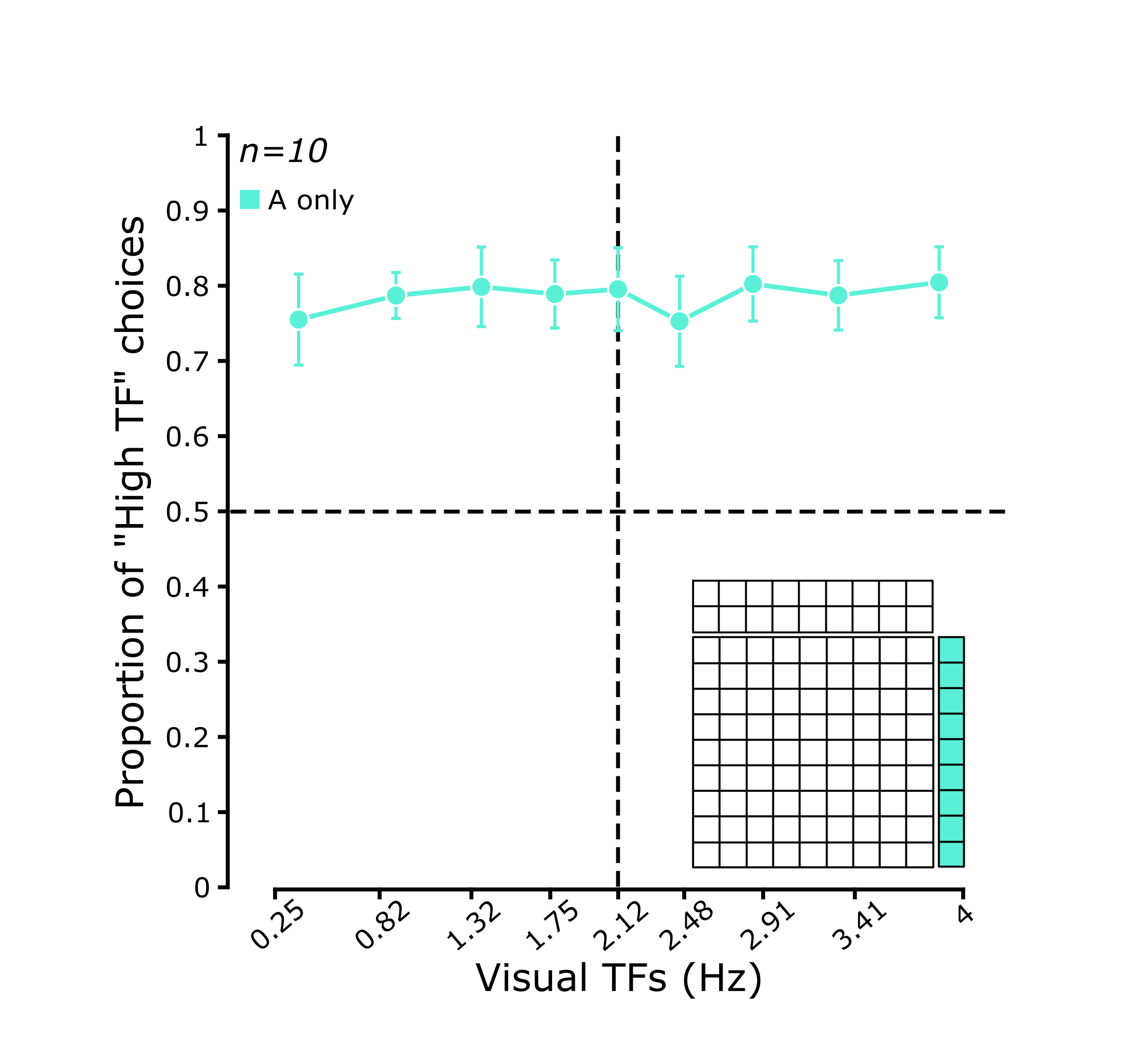

Supplement: S1 Fig — Group average proportion of “High TF” choices (n = 10 rats) as a function of the TF of the unimodal auditory stimuli. Error bars are SEM. (PNG) [file pcbi.1013608.s001.png]

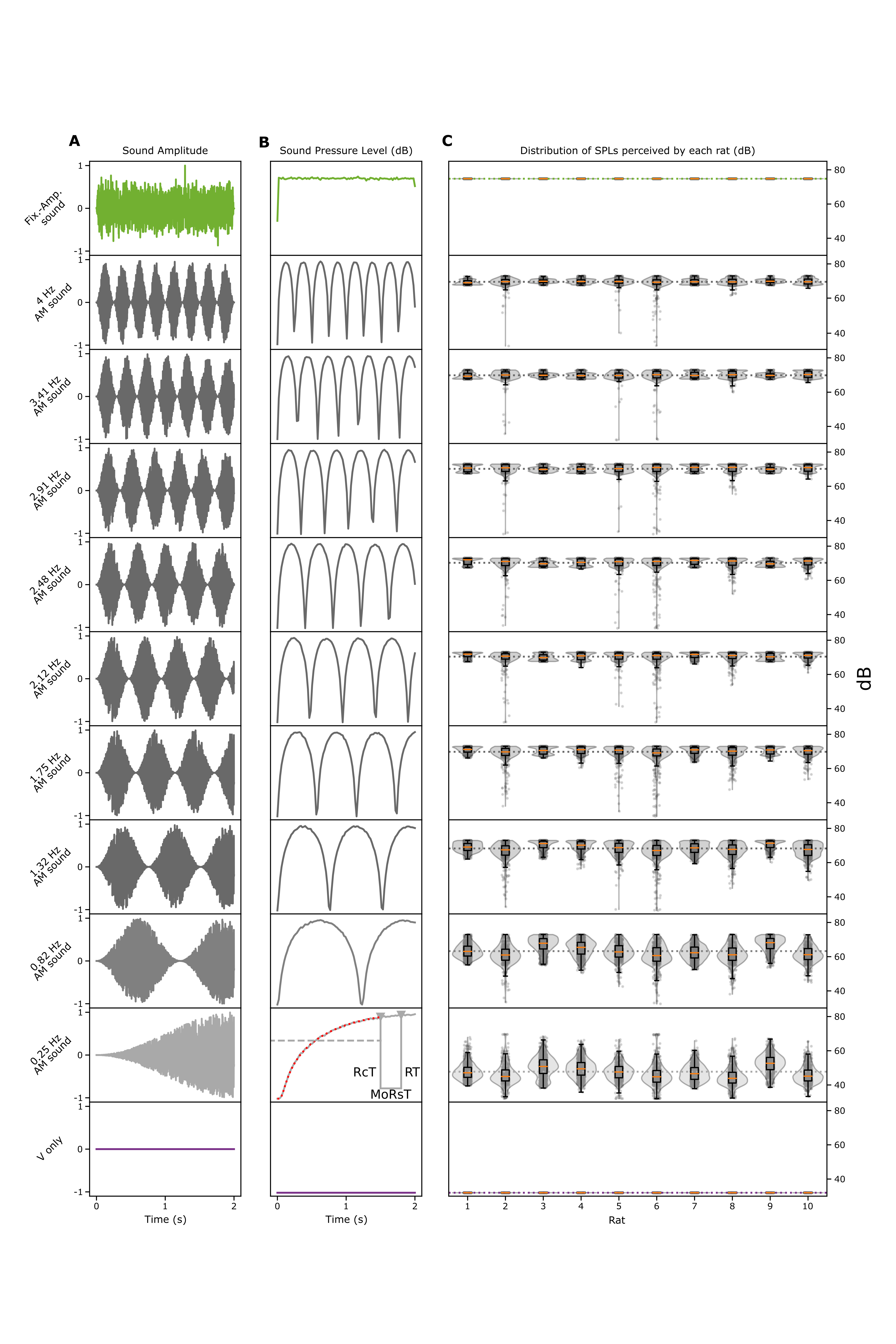

Supplement: S2 Fig — Each row refers to a distinct auditory stimulus used in the experiment: 1) the white noise burst with fixed maximal amplitude (top row; green); 2) the amplitude-modulated white noise bursts, with the 9 different temporal frequencies of the sinusoidal envelops (middle rows; gray); and 3) the absence of the auditory stimulus (bottom row; purple). A. Waveforms of the auditory stimuli used to drive the speakers that delivered the sounds to the rats (note that, for the sake of visualization, the sounds are not shown at their actual sampling frequency of 44.1 KHz but they have been downsampled to 500 Hz). B. Intensities of the auditory stimuli in dB as a function of time, as computed based on the waveforms shown in A and the measured intensity of 10 s long white noise bursts with different amplitudes (see the Materials and Methods for details). The panel referring to the TF of 0.25 Hz illustrates how the average sound intensity experienced by a rat in a given trial was computed, based on the reaction time RcT of the animal. The latter was obtained by subtracting the estimated motor response time MoReT to the measured response time RT. C. Distributions and estimates of the average sound intensities experienced by each rat across all trials recorded for any given stimulus condition. Scatter plots show the estimated sound intensity experienced by the rats in each individual trial. Violin plots represent the full probability density distributions of these estimates while box plots show their median and interquartile range. The dashed line indicates the across-rat average intensity per stimulus condition. (PNG) [file pcbi.1013608.s002.png]

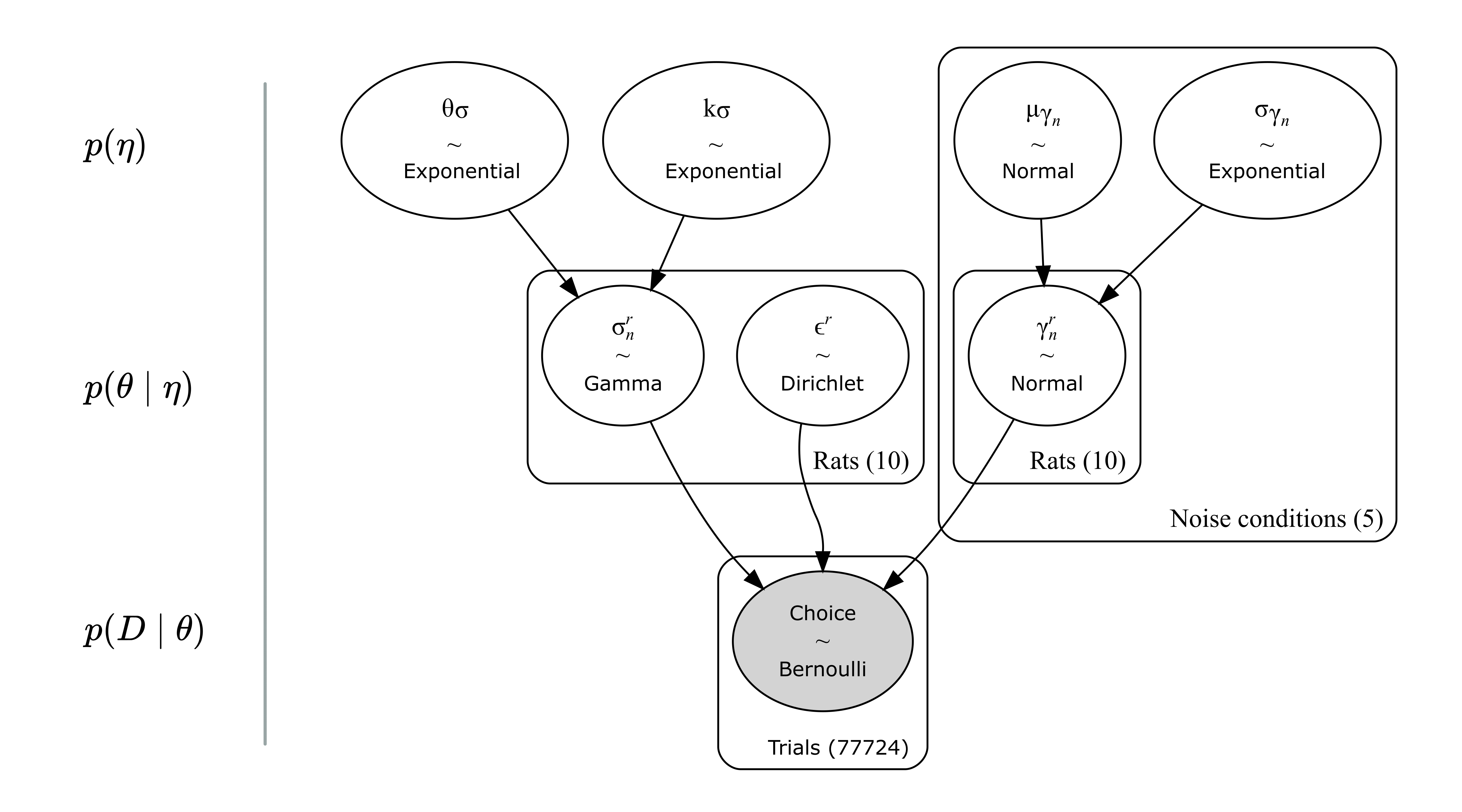

Supplement: S3 Fig — On the left, each row is labeled as representing the population parameters priors (p(η)), the subject-level parameters priors (p(θ∣η)) or the likelihood (p(D∣θ)). On the right, each bubble represents a parameter of the model. The name of the parameter and its distribution are reported. The rectangular plates indicate that multiple iid variables have been grouped. The group name and the corresponding number of variables are indicated on the bottom left of each plate. Each arrow represents the dependencies of the model. Gray coloring indicates that the variable is conditioned to observations. (PNG) [file pcbi.1013608.s003.png]

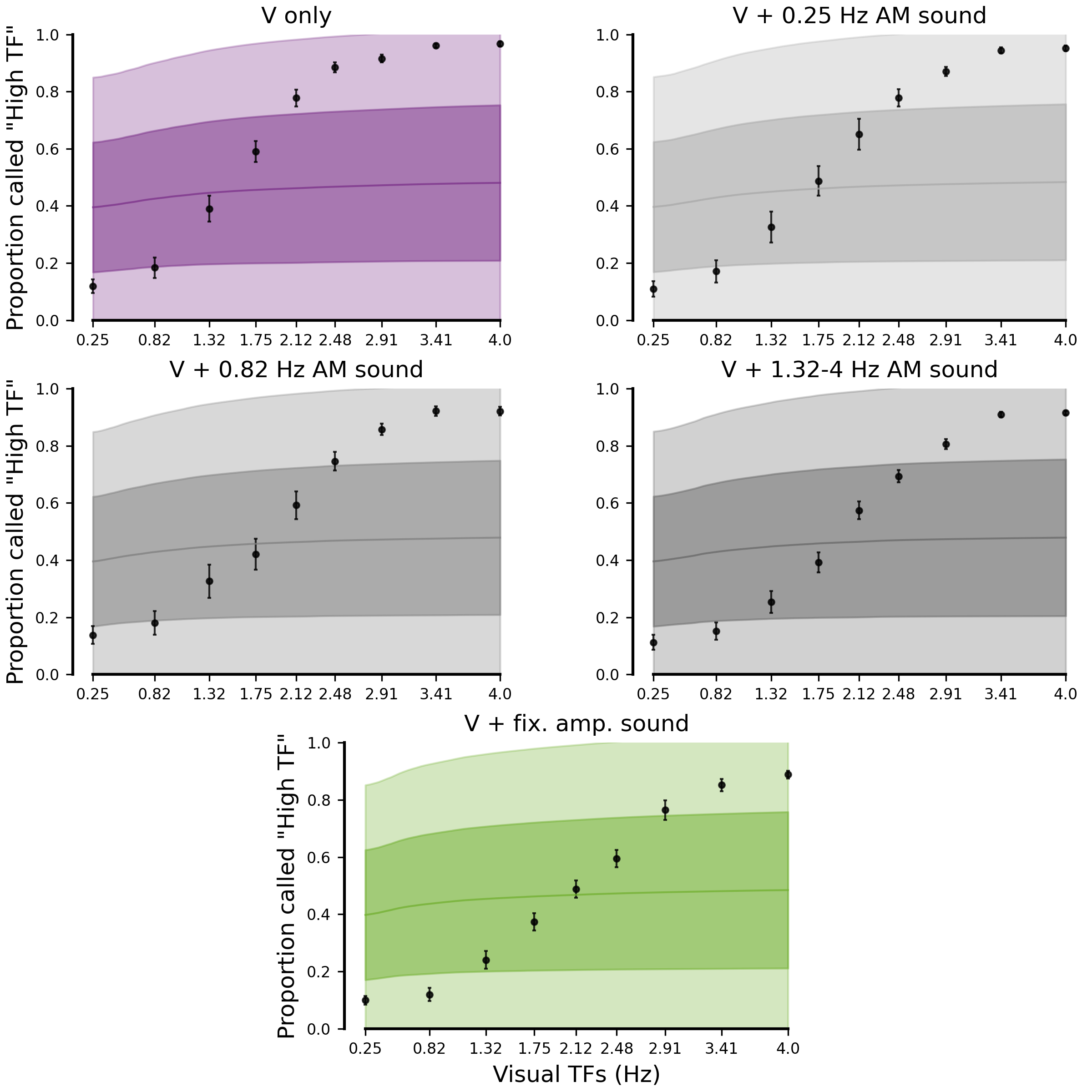

Supplement: S4 Fig — In black the average proportion of answers "High" of the rats. Error bars denote the s.e.m. across all rats. Each colored line is the average predicted psychometric curve obtained for one of the five noise condition, generated by parameters sampled from its respective prior distributions. The γ and σ parameters are drawn from the population average parameters; the ϵ parameters, given the absence of a hierarchical structure, are averaged across all rats. The shaded areas represent one (dark area) and two (dim area) standard deviations from the mean. The five curves all look almost identical (up to sampling noise) as the priors for the noise conditions were the same. (PNG) [file pcbi.1013608.s004.png]

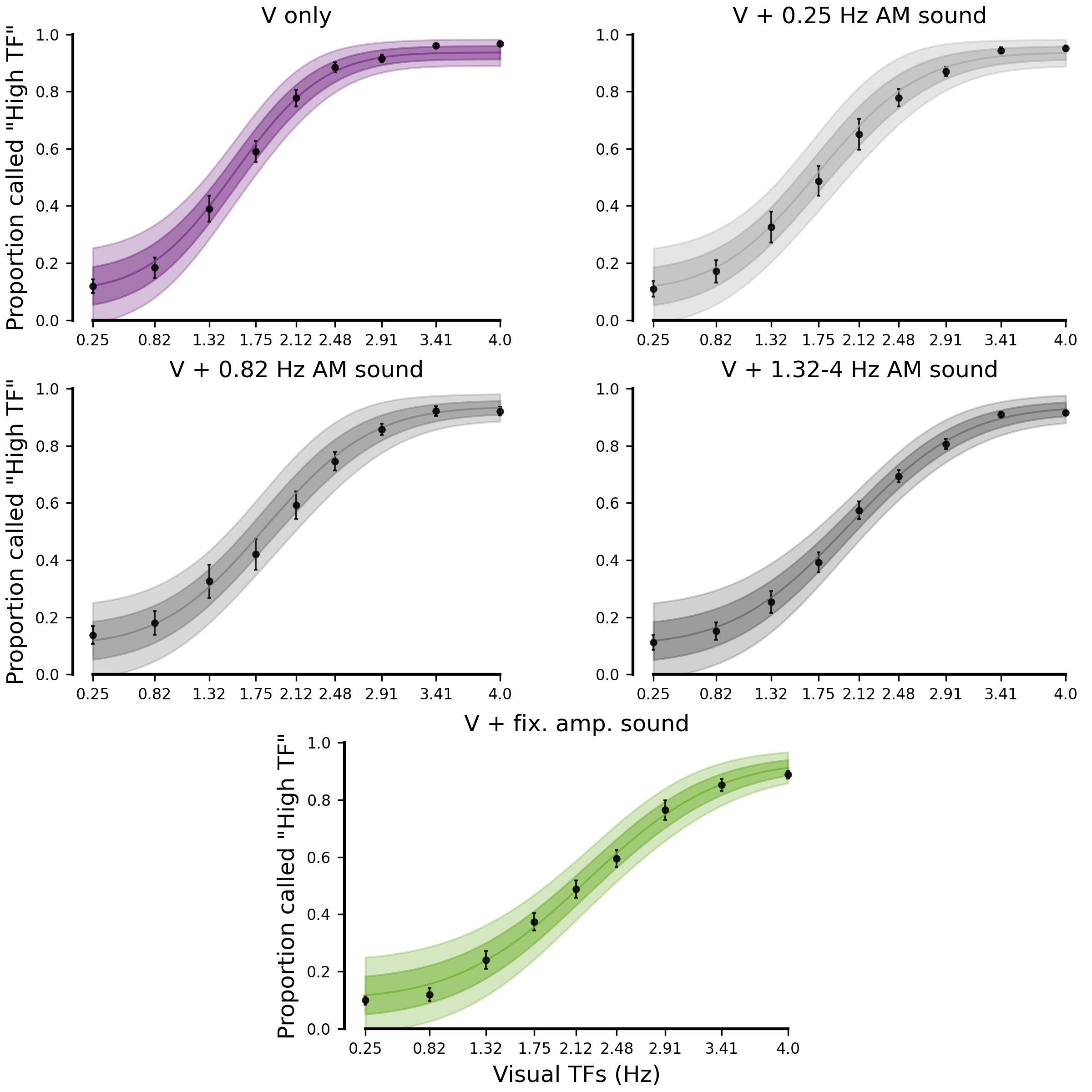

Supplement: S5 Fig — In black the average proportion of answers "High" of the rats. Error bars denote the s.e.m. across all rats. Each colored line is the average predicted psychometric curve obtained for one of the five noise condition, generated by the parameters sampled in the MCMC chain, following the respective posterior distributions (same as the solid lines displayed in Fig 5A). The γ and σ parameters are drawn from the population average parameters; the ϵ parameters, given the absence of a hierarchical structure, are averaged across all rats. The shaded areas represent one (dark area) and two (dim area) standard deviations from the mean of the posterior. (PNG) [file pcbi.1013608.s005.png]

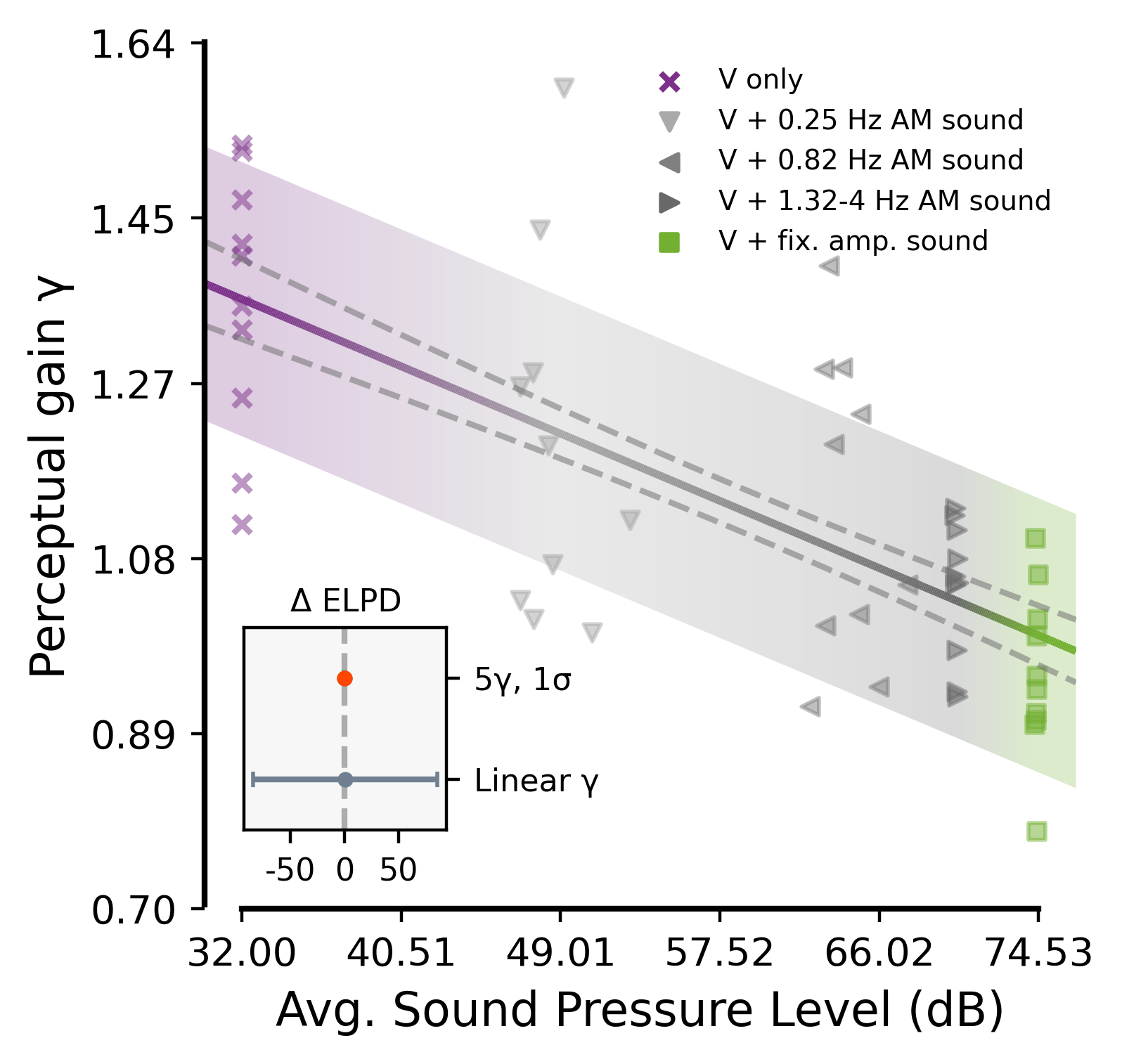

Supplement: S6 Fig — The solid line represents the population-level gain parameter μγ, modeled as a linear function of the average sound pressure level I. The slope of the line is (−8.6±1.4)×10−3, the intercept 1.636±0.083 (posterior mean ± st. dev.). The color gradient along the line maps the specific sound conditions: green for the fixed amplitude condition, grays for the V+AM conditions, and purple for the V-only condition. The dashed lines represent one standard deviation of μγ. The shaded area around the regression line represents the posterior mean of the between-subject standard deviation, σγ. This area illustrates the estimated population-level variability. The symbols (triangle, square, cross) denote the relative average perceived sound pressure (x axis) and the posterior means (y axis) for individual rats. The inset shows the relative distance in Expected Log-Predictive Density (ELPD) between the linear model and our best model (5γ, 1σ), error bars denote the standard deviation of the difference. (PNG) [file pcbi.1013608.s006.png]
