# Supplementary material for: Seeing what you hear: Compression of rat visual perceptual space by task-irrelevant sounds
Source: PLoS Comput Biol. 2025 Oct 29;21(10):e1013608. doi: 10.1371/journal.pcbi.1013608 (PMC12571299; doi:10.1371/journal.pcbi.1013608)
Supplement: Attachment — Submitted filename: PCOMPBIOL-D-25-00990.docx [file pcbi.1013608.s008.docx]

**Seeing what you hear: compression of rat visual perceptual space by task-irrelevant sounds.**

While the integration of multimodal stimuli in cortical hierarchies has been well studied, the role of how unimodal inputs shape the processing of signals carried by a different modality is less well understood. This article aimed to address this concern by investigating the impact of task-irrelevant sounds on visual perception in rats.

The study reports a behavioural training paradigm in which 10 rats (out of an initial 12) were trained to visually discriminate between drifting gratings of high and low temporal frequency (TF), while simultaneously being presented with task-irrelevant auditory stimuli. The authors tested a variety of audio-visual conditions, including: visual stimuli paired with fixed-amplitude sounds; visual stimuli with amplitude-modulated sounds (exploring various temporal frequency relationships such as congruent, incongruent, and anti-congruent); purely visual stimuli (no sound); and purely auditory stimuli (where rats’ spontaneous responses were measured without feedback). Visual stimulus contrast was progressively reduced during training, based on prior studies suggesting a stronger impact of sound on V1 responses at low visual contrast.

The key behavioural finding was that classification of visual TF was strongly and systematically altered by the presence of task-irrelevant sounds, with the effect determined by sound intensity rather than its temporal modulation.

The authors developed a Bayesian ideal observer model of rat perceptual choices, combined with a simple neural coding scheme to better understand the mechanisms underlying this phenomenon. This model framework suggests that auditory inputs induce an effective compression of the visual perceptual space. The model allowed the authors to systematically test the roles of different internal parameters: σ controlling how sound affects the sensitivity of the visual representation, and γ controlling how sound affects the gain. The study found that a model with constant visual sensitivity (a single σ parameter) but varying gain (γ parameter) across different sound intensity levels accurately captured the rats’ perceptual choices, outperforming other models with more parameters.

Overall, I believe the findings and approaches presented in the manuscript are suitable for PLOS Computational Biology as they combine behavioural experiments with computational modelling to explore biologically motivated questions, using an innovative computational framework that yields novel mechanistic hypotheses for future testing. The article also aligns with PLOS’s open science principles by providing data and analysis code on Zenodo (to be made public upon acceptance). However, the clarity of the results (specifically, the description of the behavioural findings) could be improved to make the article more accessible to non-specialist readers. These descriptions are mostly located later in the results or in the method sections, but made the initial read somewhat confusing.

Specific comments:

-The authors did a great job of simplifying the intuition behind the ideal observer model in Figure 5. I think the addition of another panel to Figure 1 (pg 5) could similarly help simplify the logic of the behavioural results by highlighting two key points that are important for interpretation: 1) that both temporal frequency and intensity differ in auditory stimuli, and 2) that training occurred specifically with fixed amplitude sound.

Forgive the rough sketch, but my suggestion would be to include a new panel in Figure 1C showing the mean ± SEM of estimated sound intensity across rats for each auditory frequency, as well as for the visual-only and fixed-amplitude sound conditions. The current panel C could then be moved to D.

In this new panel, the colour coding could help establish the conventions used throughout the manuscript (purple for the visual-only, green for the fixed-amplitude sound and with different shades of grey to represent sound intensity levels, reinforcing the idea that grey indicates sound intensity (not temporal frequency) in the subsequent plots. You could also consider adding a few small insets showing some example waveforms. Additionally, I would recommend specifying in the axis label that rats were trained under the fixed-amplitude sound condition. This, in combination with your current Figure 1, would provide readers with a clearer and more intuitive understanding of the experimental design and stimuli right from the start.

-Other than mentioning the green trace on page 5, Figure 2 is not referenced again until much later in the results/methods. With the additional panel in Figure 1, Figure 2 could potentially be moved to later in the Results section (eg. from pg 7 to pg 12) or included in the Supplemental Material.

-pg 6 line 79 “Two of the rats failed go beyond Training Phase 2 and were therefore

excluded from further testing”, I suggest rewording this to be more explicit eg. Two of the rats failed to achieve the performance criteria in Training Phase 2 after x sessions and were therefore excluded from further testing.

-pg 7. Figure 2 caption C) could use more detail. eg. Scatterplots show individual trials; violin plots illustrate the distribution of estimated intensities for each sound condition and rat; the dashed line indicates the across-rat average intensity.

-pg 9 Figure 3 in the legend for visual contrast, there are 4 levels of grey (75, 50, 35, 25) but in the colour labelling of the training sessions axes only 3 levels are shown. Maybe this is a pdf conversion error, but please double-check and adjust to ensure consistency.

Similarly, rat 1 doesn’t seem to have any green segments on the line plot, does this mean they never had training phase 4? Or the green line segment lost. Finally, please clarify what the grey segments of the line plot indicate?

-pg 10 Figure 4 caption. I suggest removing the detailed description of the ANOVA here “Table 1 reports the results of a 2-way ANOVA having as factors the experimental condition (“V + fixed amplitude sound”, “V + AM congruent” and “V + AM anti-congruent”) and the grating TF, as well as the results of a post-hoc Tukey test between each pair of experimental conditions”. Instead, a more concise statement would be sufficient eg. Statistical comparisons are reported in Tables 1 and 2.

Conversely, the table captions benefit from additional detail eg. Repeated-measures two-way ANOVA on the proportion of “High” visual stimulus responses, and including descriptions of Visual TF levels and Experimental Condition levels for each case.

-pg 14 Figure 5A) Minor detail, but consider adding a colour gradient to the sound intensity arrow for consistency with how sound intensity is represented throughout the results.

-pg 30. It is not entirely clear from the text during which training phases the contrast reduction occurred. Did this only happen after Phase 5, once all temporal frequencies had been introduced?

Additionally, more detail on the structure of training and testing sessions would be helpful. eg. What was the trial structure for both training and testing sessions? Over how many sessions was the testing phase conducted? Did each testing session include all combinations of the experimental conditions? Was there a typical number of trials per session, or was this determined by the behaviour of the rat on a given day etc? Clarifying these would improve the reproducibility of the experimental design.
