# Supplementary material for: Seeing what you hear: Compression of rat visual perceptual space by task-irrelevant sounds
Source: PLoS Comput Biol. 2025 Oct 29;21(10):e1013608. doi: 10.1371/journal.pcbi.1013608 (PMC12571299; doi:10.1371/journal.pcbi.1013608)
Supplement: Attachment — Submitted filename: Zoccolan_response_to_reviewers.docx [file pcbi.1013608.s009.docx]

**Point-by-point response to the reviewers:**

**PLOS Computational Biology - PCOMPBIOL-D-25-00990**

We thank the reviewers for their careful consideration of our manuscript. We have revised our manuscript to take into account the points raised in the reviews, by rewriting or expanding several passages, updating three main figures **(Figures 1, 2 and 4)**, adding one main figure **(new Figure 6),** adding four supplementary figures **(new supplementary figures S2, S4, S5, S6)** and adding one supplementary table **(new supplementary table S1)**. All altered passages are now highlighted in **green** in the marked-up copy of our manuscript. We also reply below in detail to each of the reviewers’ points (our answers are highlighted in green).

# Reviewer 1

... Overall, I believe the findings and approaches presented in the manuscript are suitable for PLOS Computational Biology as they combine behavioural experiments with computational modelling to explore biologically motivated questions, using an innovative computational framework that yields novel mechanistic hypotheses for future testing. The article also aligns with PLOS’s open science principles by providing data and analysis code on Zenodo (to be made public upon acceptance). However, the clarity of the results (specifically, the description of the behavioural findings) could be improved to make the article more accessible to non-specialist readers. These descriptions are mostly located later in the results or in the method sections, but made the initial read somewhat confusing.

We thank the reviewer for the overall positive appraisal of our work. We answer the specific clarity suggestions below.

## Specific comments:

-The authors did a great job of simplifying the intuition behind the ideal observer model in Figure 5. I think the addition of another panel to Figure 1 (pg 5) could similarly help simplify the logic of the behavioural results by highlighting two key points that are important for interpretation: 1) that both temporal frequency and intensity differ in auditory stimuli, and 2) that training occurred specifically with fixed amplitude sound.

Forgive the rough sketch, but my suggestion would be to include a new panel in Figure 1C showing the mean ± SEM of estimated sound intensity across rats for each auditory frequency, as well as for the visual-only and fixed-amplitude sound conditions. The current panel C could then be moved to D.

In this new panel, the colour coding could help establish the conventions used throughout the manuscript (purple for the visual-only, green for the fixed-amplitude sound and with different shades of grey to represent sound intensity levels, reinforcing the idea that grey indicates sound intensity (not temporal frequency) in the subsequent plots. You could also consider adding a few small insets showing some example waveforms. Additionally, I would recommend specifying in the axis label that rats were trained under the fixed-amplitude sound condition. This, in combination with your current Figure 1, would provide readers with a clearer and more intuitive understanding of the experimental design and stimuli right from the start.

We thank the reviewer for the great suggestion. We agree that adding such schematic will make the paper easier to read. As suggested, we have added this schematic as a new panel in figure 1 **(Figure 1D).**

-Other than mentioning the green trace on page 5, Figure 2 is not referenced again until much later in the results/methods. With the additional panel in Figure 1, Figure 2 could potentially be moved to later in the Results section (eg. from pg 7 to pg 12) or included in the Supplemental Material.

We agree with this suggestion and we have decided to move old Figure 2 to the Supplementary Material (the figure is now named **Supplementary Figure S2**).

-pg 6 line 79 “Two of the rats failed go beyond Training Phase 2 and were therefore excluded from further testing”, I suggest rewording this to be more explicit eg. Two of the rats failed to achieve the performance criteria in Training Phase 2 after x sessions and were therefore excluded from further testing.

We have changed the sentence as suggested by the reviewer (see **page 6 of our revised Results** section).

-pg 7. Figure 2 caption C) could use more detail. eg. Scatterplots show individual trials; violin plots illustrate the distribution of estimated intensities for each sound condition and rat; the dashed line indicates the across-rat average intensity.

We thank the reviewer for the suggestion; we have revised the legend accordingly (as per your other suggestion above, the figure has now been moved to the supplement and is now **Supplementary Figure S2**).

-pg 9 Figure 3 in the legend for visual contrast, there are 4 levels of grey (75, 50, 35, 25) but in the colour labelling of the training sessions axes only 3 levels are shown. Maybe this is a pdf conversion error, but please double-check and adjust to ensure consistency.

We thank the reviewer for noticing the issues with Figure 3 (which has now become **the new Figure 2** in our revised manuscript). Upon revising the figure, we realized that the inconsistency did not stem from a pdf conversion error, but from a mistake we made in selecting the sessions that had been plotted in the figure. In fact, the original figure showed only a portion of the sessions recorded during training phases 2-5 and only a portion of the sessions where contrast was gradually reduced. In addition, although these portions were not consecutive, their sessions had been erroneously collated. These inconsistencies only concerned the details of the training procedure of the rats and did not affect any of our results or their interpretation. However, it is very important to us that our experiments are described to the highest standards of transparency and reproducibility, and, therefore, we are particularly grateful to the reviewer for asking the question that led us to discover these issues. We have now corrected these inconsistencies and we have produced a new version of the figure that tracks the progression of rat accuracy across a larger stretch of sessions, from the beginning of phase 2 to the end of the contrast reduction procedure.

Contextually, we have also improved and expanded the description of the contrast reduction procedure (see **pages 6-7 of our revised Results section and pages 31-32 of our revised Methods section**). Here we explain how this procedure was actually run two times for each rat. A first, exploratory contrast reduction phase was initially run to quickly reduce the contrast of the gratings within a few sessions (occasionally bringing the contrast down to 15% for the top performing animals; see rat 1 in our **new Fig. 2A**). We then brought back the contrast to 100% to make sure that the animals had not simply adapted to a restricted range of contrasts, but had instead acquired a stable and generalizable strategy for solving the classification task across the full spectrum of contrasts. We finally performed a second contrast reduction to bring the contrast of the gratings to the final, stable value of 25%, to be used in the experiments of the test phase. For some animals, the first contrast reduction phase resulted in a substantial drop of the performance (see rat 2 in **new Fig. 2B**, session 39). In these cases, the contrast reduction procedure was stopped and the contrast was raised back to 100%, to allow the animal to attain again a stable accuracy >70% at full contrast (sessions 40-46). Following this recovery period, the contrast reduction procedure was resumed (47-54).

Importantly, although the time course of the contrast reduction phase varied across animals (depending on their proficiency in the task), the final outcome was consistent and robust: in all cases, the rats reached stable performance > 70% correct at 25% contrast across visual temporal frequencies, which defined the entry point to the testing phase.

Similarly, rat 1 doesn’t seem to have any green segments on the line plot, does this mean they never had training phase 4? Or the green line segment lost. Finally, please clarify what the grey segments of the line plot indicate?

We apologize for the confusion. In the new version of the figure (**Fig. 2**), the accuracy plot now represents the various training phases with colored markers instead of continuous colored segments, thus providing a clearer and more robust visual interpretation. Markers more appropriately depict training sessions as discrete events, whereas continuous segments could misleadingly suggest that performance was measured continuously across phases. In the original version of the figure, the grey lines resulted from connecting transitions between different training phases. This issue has now been corrected, and the revised figure avoids any such ambiguity.

-pg 10 Figure 4 caption. I suggest removing the detailed description of the ANOVA here “Table 1 reports the results of a 2-way ANOVA having as factors the experimental condition (“V + fixed amplitude sound”, “V + AM congruent” and “V + AM anti-congruent”) and the grating TF, as well as the results of a post-hoc Tukey test between each pair of experimental conditions”. Instead, a more concise statement would be sufficient eg. Statistical comparisons are reported in Tables 1 and 2.

Conversely, the table captions benefit from additional detail eg. Repeated-measures two-way ANOVA on the proportion of “High” visual stimulus responses, and including descriptions of Visual TF levels and Experimental Condition levels for each case.

Thank you for your suggestion. We have now moved the details about the ANOVA analysis from the **legend of Fig. 3** (i.e., former Fig. 4) **to the legends of Tables 1 and 2**.

-pg 14 Figure 5A) Minor detail, but consider adding a colour gradient to the sound intensity arrow for consistency with how sound intensity is represented throughout the results.

This is an excellent suggestion, which we have implemented in **our new Fig. 4A** (i.e., former Fig. 5A). We have adopted the same visual language also in the **new Supplementary Figure S6**.

-pg 30. It is not entirely clear from the text during which training phases the contrast reduction occurred. Did this only happen after Phase 5, once all temporal frequencies had been introduced?

Yes, the contrast was reduced right after Phase 5. We have now updated the text to make this passage clearer (see **page 31 in our revised Methods section**).

Additionally, more detail on the structure of training and testing sessions would be helpful. eg. What was the trial structure for both training and testing sessions? Over how many sessions was the testing phase conducted? Did each testing session include all combinations of the experimental conditions? Was there a typical number of trials per session, or was this determined by the behaviour of the rat on a given day etc? Clarifying these would improve the reproducibility of the experimental design.

We have expanded our **revised Methods section** to include more details on the structure of the training and testing sessions **(see pages 31-33).** As already mentioned, we have also updated the figure illustrating the training history of the two example rats (i.e., the former Fig. 3, **which is now Fig. 2**) by providing a more complete overview of the rats’ performances over the training days, along with the number of trials the animals completed in each session.

Concerning the specific questions of the reviewer:

1. Regarding the trial structure, this is explained in detail at the beginning of the “Behavioral task” section in the Methods section (see **page 29**). Here we list all the events that take place during a trial (i.e., the triggering of stimulus presentation by the rat, the way in which the animal delivers his behavioral choices, the way the audiovisual stimuli are presented, the way in which reinforcement is provided, etc.). This trial structure was the same for all the training and testing sessions (we now state this more clearly **on page 29 of our revised Methods section**).
2. Regarding the question about the number of sessions and the number of trials per sessions, we now specify these details for both the training phase (**revised Methods section, pages 31-32**) and the test phase (**revised Methods section, page 32-33**; here we also specify that all stimulus conditions shown in Fig. 1C were presented in each test session).

# Reviewer 2

In this study, the authors trained a group of rats in a visual temporal frequency classification task, in which visual stimuli were presented simultaneously with, but were task-irrelevant to, auditory stimuli. They developed a Bayesian ideal observer model, which captured the full spectrum of the rats’ perceptual choices. By integrating psychophysical experiments with computational modeling, the study identifies inhibition as a key mechanism underlying auditory–visual interactions.

Below are my comments:

(1) The authors employ a Bayesian ideal observer model, which is a commonly used approach. To improve clarity, it would be useful to specify whether any adaptations were made to the conventional model to suit the current experimental design.

Ideal observer modeling is indeed a very popular approach. Stated simply, an ideal observer is the optimal way of solving a given perceptual task, under a specific set of assumptions about the perceptual process and a specific notion of optimality. While there are standard methodologies for the development of ideal observers, there is strictly speaking no “conventional model”, because each behavioral task will necessarily lead to a different observer/model (or, more correctly, to a family of different observers, each arising from different assumptions about the details of the sensory process). However, as the reviewer points out, it can be useful for the reader to be able to connect our model to other, similar models they may have seen in the literature. In our case, the main feature distinguishing our task from a standard binary sensory classification experiment is the presence of the task-irrelevant auditory signals. In the ideal observers we develop in the present work, we chose to model the effect of these auditory signals as a change in the gain of the (linear) neural representation of the visual stimuli. In this sense, our model resembles a type of observer commonly used for binary classification tasks, with the variable gain being the main difference. We now explain this better in the text, where we have also added more references to prior works (see **page 34 of our revised Methods section**).

(2) To validate the modeling approach, the authors should present goodness-of-fit measures that quantify the agreement between the model predictions and the observed behavioral data.

We thank the reviewer for this suggestion. As a simple measure of goodness-of-fit, we have calculated the R squared for our best model, computed over the population-level psychometric curves, to capture the level of agreement between data and model prediction in **Fig. 5** (old Fig. 6). The R squared is 0.9973 ± 0.0002 (posterior mean ± standard deviation). We have now reported this information **in the legend of Fig. 5 (page 16 of our revised Results section)**.

(3) The authors are encouraged to provide a clear summary of the critical model parameters, as this would significantly improve the transparency, reproducibility, and understanding of the modeling outcomes.

We thank the reviewer for this suggestion. We have included a new table which summarizes the inferred values of the main parameters of the model as well as a battery of standard diagnostics for MCMC-based Bayesian inference (**Supplementary Table S1**). This will make it much easier for the interested reader to – for instance – code a simple simulation that behaves like the “average rat” in our dataset. We also note here that the data that we will publish alongside of the paper (currently available through the private Zenodo link given in the Data Availability Statement) includes the PyMC code for our models as well as the full Markov Chain used to derive the results presented in our paper. This makes it straightforward to reproduce our statistical results, repeat our inference, or compute arbitrary statistics for other quantities of interest.

(4) The model's three underlying assumptions are outlined by the authors; however, further justification through citations from existing literature would enhance the theoretical foundation and clarify the rationale behind each assumption.

Following the suggestion of the reviewer, we have added a justification for each of the three assumptions at the base of our model, including references to the relevant literature. See our **revised Results on pages 11-12**.

(5) In Figure 3, while rat 2 begins with a relatively high discrimination accuracy (~0.8), a noticeable decline occurs around training session 27, differing markedly from the trend observed in rat 1. The authors should provide a rationale for this discrepancy.

We understand the concern of the reviewer. However, it is far from surprising for rats to display sudden (but temporary) drops of performance (lasting 2-3 sessions), especially when they are learning a new task. These drops are idiosyncratic (not all rats show them and, when drops occur, they do not happen at the same epoch of the training for every animal). The causes of a specific drop are hard to infer, since they likely reflect fluctuations in the level of motivation, deployment of exploratory strategies, or temporary failures of the behavioral rig (e.g., uneven delivery of liquid from the reward ports). Because of this, these drops are often accompanied by a marked reduction in the number of trials completed by the animal in the corresponding sessions (this can now be appreciated in **our revised Fig. 2**, where a bar plot shows the number of trials completed by a rat across the training sessions). Regardless of the cause, such drops are not worrisome as long as they are temporary (as for example rat 2 in our former Fig. 3, **now Fig. 2**) and the animal promptly regains its setpoint performance level in a few sessions. We have added an explanation to illustrate what stated above at **page 6 of our revised Results section**.

Please also note that we realized we made a mistake in the plots previously shown in Fig. 3 (**now Fig. 2**). Only some portions of the training sessions had been plotted and these portions had been collated despite not being contiguous. In our revision, we have corrected these inconsistencies and we have fully revised this figure, which now shows all the sessions of training phases 2-5 and of the contrast reduction procedure (which was actually repeated in multiple bouts; see **our revised Method section at pages 31-32**). This now provides a complete description of the training history of the two example rats (for instance, it allows appreciating that, at the beginning of phase 2, both rats started at chance and then their performance quickly raised to >70% correct within 6-7 sessions).

(6) The identification of inhibition as a key mediator of auditory–visual interactions represents a core conclusion of this study. To enhance conceptual clarity, the authors could consider providing a schematic diagram that illustrates the proposed inhibitory mechanism and its role in cross-modal processing.

We thank the reviewer for this great suggestion. We have added a **new figure (Fig. 6)** with an outline of the hypothesized interaction between auditory and visual cortex underlying the sound-driven compression of rat visual perceptual space. The figure is described at the end of our **revised Discussion section** (**page 22**).

# Reviewer 3

It was a joy for me to read this excellent paper by Zanzi and Rinaldi et al. They report a carefully study on the effect of simultaneous task-irrelevant auditory stimulation on performance in a visual two-alternative forced choice discrimination task in rats. They find that auditory stimulation compresses the visual perceptual space in rats, and that increasing auditory intensity leads to increasing visual perceptual space compression. With impressive thoroughness, they construct a computational model that accounts for the observed data in a clear and straightforward manner. This study provides answers to several open questions about the nature of 'horizontal' (in terms of an inferential hierarchy) cortical connectivity between primary sensory cortical regions, and it clearly refutes some of the speculative explanations offered for observations from previous studies. In particular, Zanzi and Rinaldi et al. find that it is the total intensity of sensory stimulation prior to decision-making that determines the degree of compression of visual perceptual space, and more fundamentally that it is this compression which gives rise to the observed phenomena.

We thank the reviewer for the positive assessment of our work. We are glad it was an enjoyable read.

Since this study is extremely well-conceived and executed, and since the attendant modelling is entirely convincing, I believe it can be pubished more or less as submitted. Nonetheless, I have the following remarks:

- Looking at Figure 6B, wouldn't it have made sense to have a linear model (two parameters: intercept and slope) for the dependency of gamma on average sound pressure level instead of fitting a number of independent values for gamma and then embarking on a big model comparison exercise?

We thank the reviewer for this suggestion. In the present work, we focused on showing that the neural gain in visual cortex depends on the sound intensity, and we had no particular hypothesis to justify a linear dependence. A model like the one we used (where gamma is free to take a different value for each value of sound intensity, or for each group of values) has fewer constraints than a linear model and is, in our opinion, better suited to the task as it allows in principle to capture other types of relationships between sound intensity and gain. However, we agree that, a posteriori, the relationship highlighted in old Fig. 6B (now **Fig. 5B**) suggests that a linear dependence should be a “good enough" description. We have therefore added a supplementary figure **(Supplementary Figure S6)** showing that indeed the relationship between sound intensity and gain can be well described by a linear relationship. A description of this figure is provided at **page 17 of our revised Results section**.

- While quantitative model comparisons of the kind reported can sometimes be helpful, it is generally more informative to choose a model on the basis of its performance in prior and posterior predictive simulations. It would therefore be interesting to see prior predictive simulations in addition the posterior ones in Figure 6A.

We thank the reviewer for this suggestion. We have now added two supplementary figures, containing:

- prior predictive simulations showing the range of behaviors implied by our model and related priors **(Supplementary Figure S4).** These are described at **page 43 of our revised** **Methods section**.
- more detailed posterior predictive simulations **(Supplementary Figure S5)**. This figure expands on Fig. 5A by showing the uncertainty associated with each population-level psychometric curve, and offers a direct comparison with the prior predictive plots. Details on the posterior predictive simulations are provided at **page 44 of our revised** **Methods section**.

- Reinforcing the previous point, the priors of the chosen computational model should be justified by prior predictive simulation instead of an appeal to convention (line 857). Reference 54 (to which you appeal) would agree.

We agree; indeed, the prior predictive simulations mentioned at the previous point highlight that our assumed priors are flexible enough to allow a full range of behavior for the psychometric curves, and have no bias towards supporting the central claim of our paper, namely the dependence of the gain on the sound intensity.

- Given the authors' impressive modelling capabilities, I was surprised to find some of the data analyzed by ANOVA, an unregularized out-of-the-box procedure.

We feel that providing an initial analysis of the behavioral data with a simple ANOVA will make it easier for readers with a less technical background to follow the narrative of the paper, without getting immediately distracted by the details of our statistical analysis. At the same time, we feel that nothing is lost for the more statistically savvy reader, as the Bayesian modeling results (**Fig.5**) fully subsume and confirm those presented in Fig. 3 and Tables 1 and 2.
